# Cytological investigations and new chromosome number reports in yarrow (*Achillea millefolium* Linnaeus, 1753) accessions from Iran

**DOI:** 10.3897/CompCytogen.v7i4.6075

**Published:** 2013-10-24

**Authors:** Fatemeh Afshari, Mohsen Ebrahimi, Mohammad Akbari, Mostafa Farajpour

**Affiliations:** 1 Biology Department, Faculty of Science, Payam Noor University, Tehran, Iran; 2 Department of Agronomy and Plant Breeding, College of Abouraihan, University of Tehran, Tehran, Iran; 3 Department of Horticultural Sciences, faculty of Agriculture, University of Tabriz, Tabriz, Iran

**Keywords:** *Achillea millefolium*, karyotype, chromosome number, ploidy level

## Abstract

In this study, a new chromosome number for Iranian yarrow (*Achillea millefolium* L.) accessions was reported. Cytological analyses on four *Achillea millefolium* accessions, indicated that two accessions were diploids (2n=2x=18) and two tetraploids (2*n*=4*x*=36). Cluster analysis based on chromosomal characteristics and karyotype asymmetry, categorized the four accessions separated into two groups. In terms of the Stebbins’ system, the karyotype of diploid accessions grouped in 2A class. The average value of the total form percentage (TF%) in the group one (diploid accessions) and two (tetraploid accessions) were 40.85 and 41.15, respectively. The group one had the highest mean value for the symmetry index (S%=57.5). Consequently, it can be inferred that diploids belonging to the group one are the earlier evolutionary forms.

## Introduction

*Achillea* is one of the most recent genera of the Asteraceae family which exists throughout the world (Rechinger 1963). More than 100 species have been identified in this genus. Many of those who used these plants reported properties such as anti-inflammatory, anti-rheumatic, antiseptic, antispasmodic, analgesic, astringent, carminative, diaphoretic, digestive, expectorant, hypotensive, stomachic and etc. (Balbir et al. 2012). These plants are native to Europe and Western Asia but are also found in Australia, New Zealand, and North America (Rechinger 1963).

*Achillea millefolium* has a high genetic and phenotypic variation in Iran (Farajpour et al. 2012, Ebrahimi et al. 2012). The basic chromosome number is often reported in different species of *Achillea* is x= 9; however, the diversity in chromosome numbers and ploidy levels are frequently occurring in the genus (Ebrahim et al. 2012). Polyploid taxa have originated in many clades including 4*x*, 6*x* and 8*x* species, and as a result, several *Achillea* species show high morphological variability (Sheidai et al. 2009). Biste (1987) explained worthy diversity in leaf width, height, shoot number, and stomata length in different populations of the same species.

In most of the chemotypes in *Achillea* sp, camphor, borneol (Rohloff et al. 2000 and Mockute and Judzentiene 2003) and 1.8-Cineole (Saeidnia et al. 2004; Barghamadi et al. 2009 and Azizi et al. 2010) have been detected. Among a number of data that can be obtained by chromosome studies: karyotype structure, karyotype asymmetry, chromosome banding, FISH, GISH and chromosome painting (Stace 2000, Levin 2002, Graphodatsky et al. 2011, Guerra 2012), the most popular, cheap and widely used approaches is that concerning karyotype asymmetry (Peruzzi and Eroğlu 2013).

*Achillea millefolium* has been cytologically analyzed extensively in different regions of the world (Felfoldy 1947, Mizianty and Frey 1973, Pireh and Tyrl 1980, Lavrenko et al. 1991, Guo et al. 2012, Bala and Gupta 2013). Three cytological studies have been reported in Iran and showed the following ploidy levels: tetraploid 2n= 4x= 36, hexaploid 2n= 6x= 54 and octoploid 2n= 8x= 72 (Farsi et al. 2000, Ebrahim et al. 2012, Sheidai et al. 2009). The aims of this study were (1) to determine the chromosome numbers of four *Achillea millefolium* accessions and (2) to find any relationship between the karyotype characteristics and asymmetrical index with ploidy levels.

## Material and methods

The aerial parts of the four *Achillea millefolium* accessions were collected from three provinces in north, west and south of Iran (Table 1). Voucher samples were deposited at the herbarium of Research Institute of Forests and Rangeland (RIFR) of Tehran, Iran. Seeds were germinated on moist filter paper in Petri dishes. Actively growing root tips, 1 to 2 cm length were cut from the germinating seeds and pretreated with 8-hydroxyquinoline (0.002M) for 2 to 4 hours and fixed in Farmer (1:3, glacial acetic acid : ethanol 95%) for 24 hours at 4° C. Thereafter, the root tips were hydrolyzed in 1 N NaOH at 60° C for 5-10 minutes, stained for 45 minutes in esterase stain at 30° C, and squashed in 45% glacial acetic acid. Finally, the chromosome images were obtained with photomicroscope.

**Table 1.** Accessions of *Achillea millefolium* studied.

**Accessions no.**	**Location**	**Latitude, Longitude**	**Elevation (m.a.s.l.)**
Am1	Iran, Ardabil, Ardabil	38°15'N, 48°17'E	1332
Am2	Iran, Ardabil, Meshkin-Shahr	37°58'N, 48°58'E	1723
Am3^†^	Iran, Ilam, Ilam	34°27'N, 46°25'E	1387
Am4	Iran, Fars, Estahban	29°12'N, 53°04'E	1767

^†^some of the characteristics of this accession were reported by Farajpour et al. (2012) in table 1 (Am30)

Karyotypec characteristics such as differences of range relative length (DRL), mean chromosome length (MCL), and mean arm ratio (MAR) were calculated using MICROMEASURE (Version 3.3) Software (Reeves 2001). Stebbin’s classification was calculated (Stebbins 1971). Cluster analysis was performed to differentiate the accessions according to the Ward’s method SPSS software for Windows 20.0 (SPSS Inc., Chicago, IL, USA).

## Results and discussion

### Karyological data

**Figure1.**
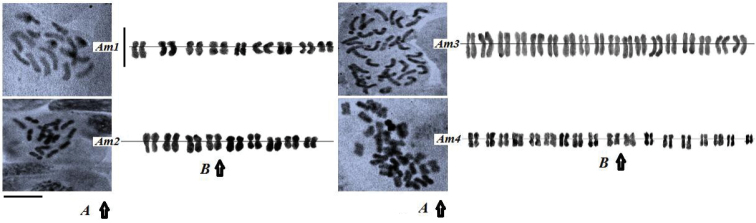
**A–B** Mitotic metaphases (**A**) and karyograms (**B**) of four *Achillea millefolium* accessions (Am1-Am4). Bars = 5μm.

Am1 and Am2 accessions were diploids (2n= 2x= 18) whereas the two other accessions (Am3 and Am4) showed tetraploid (2n= 4x= 36) level (Figure 1). According to previous studies, Farsi et al. (2009) and Khaniki (1995), reported 2*n* = 4*x* = 36 chromosomes, while Sheidai et al. (2009) and Ebrahim et al. (2012) reported hexaploid and octoploid cytotypes. In our findings, we have observed a new ploidy level (2n= 2x= 18) for two Iranian accessions of *Achillea millefolium* (Am3, Am4) that were collected in northern parts of Iran.

Karyotypic analysis indicated asymmetrical pattern in the four accessions of *Achillea millefolium* (Table 2). Mitotic metaphases and karyograms of the four accessions are shown in Figure 1. The highest TCL value was found in Am3 (60.9 µm) and the lowest was found in Am2 (24.5 µm) (Table 3). The lowest and the highest DRL values were found in Am3 and Am2 accessions, respectively (Table 3). High DRL value leads to more changes in the construction of chromosomes. DRL values in the two diploid accessions were higher than the tetraploid ones; it can be a relationship between ploidy level and DRL value. The tetraploid accessions had the most symmetric karyotypes.

**Table 2.** Karyotype features of four *Achillea millefolium* accessions.

**Accession**	**2*n***	**Ploidy level**	**TF%**	**S%**	**Karyotype formulae**
Am1	18	2*x*	40.9	64	1M+8m
Am2	18	2*x*	40.8	51	1M+6m+2sm
Am3	36	4*x*	40.5	63	16M+2sm
Am4	36	4*x*	41.8	37	17M+1sm
Mean ofgroup1	-	-	40.85	57.5	-
Mean ofgroup2	-	-	41.15	50	-

^‡^ TF%=total form percentage (sum of short arm lengths of individual/total haploid length of the complement chromosomes), S% -symmetry index (shorter chromosome length / longer chromosome length), karyotype formula (m, median region; sm, submedian region; M, median point).

**Table 3.** Total chromosome length (TCL), mean chromosome length (MCL), mean arm ratio (MAR), difference of range relative length (DRL), chromosome length range (CLR), Symmetry Classes of Stebbins (SC) of four *Achillea millefolium* accessions.

**Accession**	**TCL( µm)**	**MCL (µm) (±SD)**	**MAR( µm)**	**DRL (µm)**	**CLR (µm)**	**SC**
Am1	26.4	2.93(±0.13)	0.71	4.92	2.4-3.7	2A
Am2	24.5	2.72(±0.17)	0.69	6.9	1.8-3.5	2A
Am3	60.8	3.37(±0.09)	0.68	2.2	2.6-4.1	1A
Am4	41	2.27(±0.11)	0.73	4.49	1.1-2.9	1B

Other parameters that indicate karyotype asymmetry are total form percentage (TF %; Huziwara 1962) and symmetrical index (S% or S/L%; Battaglia 1955) (Table 2). Group one (Am1 and Am2) had the highest mean value for the symmetrical index (S%=57.5) than the group two (S%= 50) (Table 2). It can be inferred that the group one, as diploids, are the earlier evolutionary form. The average value of the total form percentage (TF%) in the group one and two were 40.85 and 41.15, respectively. The TF% index has frequently been used to explain karyotype asymmetry (Mercado-Ruaro and Delgado-Salinas 1998, Ruas et al. 2000). In terms of the Stebbins’ system, the karyotype of Am1 and Am2 grouped in 2A class, and it can be ancient evolutionary origin of *Achillea millefolium* species.

### Cluster Analysis

**Figure 2.**
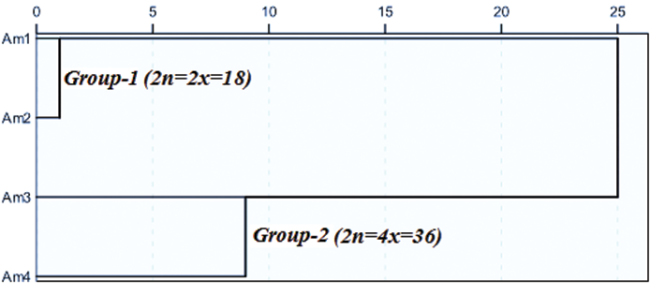
Dendrogram of four *Achillea millefolium* accessions based on the karyotype characteristics and asymmetry.

Cluster analysis was done based on karyotypic characteristics (TCL, MCL, MAR and DRL) and karyotype asymmetry (TF% and S%) (Figure 2) and agrees with Ebrahim et al. (2012). The results of cluster analyses divided the four accessions in two groups (Figure 2); based on ploidy levels. The first group included diploid accessions (Am1 and Am2), while the second group comprised tetraploid accessions (Am3 and Am4). In the dendrogram, distance between diploid accessions is lower than tetraploid accessions that confirm the result of Stebbins’ system that both of diploid accessions grouped in 2A class.

## Conclusion

The results of the present study illustrated a new ploidy level (2n= 2x= 18) in Iranian *Achillea millefolium* accessions. Cluster analysis indicated that accessions can be classified based on ploidy levels.
